# Association of Long‐Term BMI Change with Alzheimer‐Signature Cerebral Glucose Metabolism in Non‐Demented Older Adults

**DOI:** 10.1002/alz.090664

**Published:** 2025-01-09

**Authors:** Seunghoon Lee, Dahyun Yi, Min Soo Byun, Nayeong Kong, Joon Hyung Jung, Yoon Young Chang, Musung Keum, Hyejin Ahn, Jun‐Young Lee, Yun‐Sang Lee, Yu Kyeong Kim, Koung Mi Kang, Chul‐Ho Sohn, Dong Young Lee

**Affiliations:** ^1^ Myongji hospital, Hanyang University College of Medicine, Goyang Korea, Republic of (South); ^2^ Institute of Human Behavioral Medicine, Medical Research Center, Seoul National University, Seoul Korea, Republic of (South); ^3^ Department of Neuropsychiatry, Seoul National University Hospital, Seoul Korea, Republic of (South); ^4^ Department of Psychiatry, Keimyung University Hospital, Daegu Korea, Republic of (South); ^5^ Department of Psychiatry, Chungbuk National University Hospital, Cheongju Korea, Republic of (South); ^6^ Department of Psychiatry, Inje University Sanggye Paik Hospital, Seoul Korea, Republic of (South); ^7^ Hallym University Dongtan Sacred Heart Hospital, Hwaseong Korea, Republic of (South); ^8^ Interdisciplinary program of cognitive science, Seoul National University, Seoul Korea, Republic of (South); ^9^ Department of Neuropsychiatry, SMG‐SNU Boramae Medical Center, Seoul Korea, Republic of (South); ^10^ Department of Nuclear Medicine, Seoul National University College of Medicine, Seoul Korea, Republic of (South); ^11^ Seoul National University College of Medicine, Seoul Korea, Republic of (South); ^12^ SMG‐SNU Boramae Medical Center, Seoul Korea, Republic of (South); ^13^ Department of Radiology, Seoul National University Hospital, Seoul Korea, Republic of (South); ^14^ Seoul National University Dementia Research Center, Seoul Korea, Republic of (South)

## Abstract

**Background:**

Growing evidence suggests that low body mass index (BMI) or BMI decline from midlife is related to the risk of Alzheimer’s disease (AD) and related cognitive decline in later life. However, limited information is yet available regarding the underlying brain change that could explain such relationship. This study aims to investigate whether BMI and BMI change after midlife are associated with cerebral metabolism in late life.

**Method:**

A total of 268 non‐demented (193 cognitive normal and 75 mild cognitive impairment) older adults from 55 to 90 years of age were enrolled from the Korean Brain Aging Study for Early Diagnosis and Prediction of Alzheimer’s Disease (KBASE). Current or late‐life BMI was calculated using the measured height and body weight. Midlife (40 ∼ 50 years of age) BMI was calculated using self‐recall based midlife body weight and height. Self‐recall past body weight in cognitively healthy older adults was well‐validated^1^. All participant received [^18^F] fludeoxyglucose (FDG)‐PET at baseline. General linear model analysis was performed to assess the association between BMI and BMI changers with AD‐signature cerebral glucose metabolism (AD‐CM).

**Result:**

Midlife BMI had significant negative association with AD‐CM (p = 0.007). even after adjusting for age, sex, clinical deterioration rating (CDR) sum of box score, and apolipoprotein E ε4 positivity. Three strata of BMI change from midlife to late‐life (i.e., decreasing pattern: ≤ ‐4%, stable pattern: ‐4% < BMI change < 4%, increasing pattern: ≥ 4%) showed significantly different AD‐CM (p = 0.045). Post hoc comparison revealed that decreasing BMI pattern had significantly lower AD‐CM than increasing BMI pattern, while no difference was found between the decreasing pattern and stable pattern. Sensitivity analyses for cognitively normal participants showed similar results.

**Conclusion:**

The findings suggest a close association between long‐term BMI decline from midlife and cerebral hypometabolism in late life. Such brain hypometabolism may serve as a potential neural substrate for BMI reduction‐related cognitive decline or increased risk of AD in older adults.

**References**

1. Kyulo NL et al. (2012) Validation of recall of body weight over a 26‐year period in cohort members of the Adventist Health Study 2. Ann Epidemiol 22, 744‐746.

